# Respiratory syncytial virus infection-associated hospitalization in adults: a retrospective cohort study

**DOI:** 10.1186/s12879-014-0665-2

**Published:** 2014-12-13

**Authors:** Cheryl Volling, Kazi Hassan, Tony Mazzulli, Karen Green, Ahmed Al-Den, Paul Hunter, Rupi Mangat, John Ng, Allison McGeer

**Affiliations:** Department of Microbiology, Mount Sinai Hospital, Room 210, 600 University Avenue, Toronto, M5G 1X5 ON Canada; Department of Medicine, University of Toronto, Toronto, Canada; Department of Laboratory Medicine and Pathobiology, University of Toronto, Toronto, Canada

**Keywords:** Respiratory syncytial virus, Hospitalization, Adults

## Abstract

**Background:**

Once considered primarily a pediatric concern, respiratory syncytial virus (RSV) infection is gaining recognition as a cause of significant morbidity and mortality in adults. A better understanding of RSV epidemiology and disease in adults is needed to guide patient management and to assess the need for prophylaxis, vaccines, and treatments.

**Methods:**

We conducted a retrospective cohort study of adults admitted to four hospitals in Toronto, Canada, between September 2012 and June 2013 with RSV identified by a qualitative real-time reverse-transcriptase polymerase chain reaction assay in nasopharyngeal swab or bronchoscopy specimens. Main outcomes were hospital length of stay, need for intensive care unit (ICU) or mechanical ventilation, and all-cause mortality.

**Results:**

Eighty-six patients were identified as requiring hospitalization for RSV infection (56% female). Median age was 74 (range 19–102) years; 29 (34%) were < 65 years. Eighty-three (97%) had underlying chronic medical conditions; 27 (31%) were immunosuppressed, and 10 (12%) known smokers. The most common symptoms and signs were cough in 73 (85%), shortness of breath in 68 (79%), sputum production in 54 (63%), weakness in 43 (50%), fever in 41 (48%), and wheezing in 33 (38%). Lower respiratory tract complications occurred in 45 (52%), cardiovascular complications occurred in 19 (22%), and possible co-pathogens were identified in 11 (13%). Sixty-seven (78%) were treated with antibiotics and 31 (36%) with anti-influenza therapy. Thirteen (15%) required ICU care and 8 (9%) required mechanical ventilation. Five (6%) died during hospitalization. Need for ICU and mechanical ventilation were associated with mortality (P ≤ 0.02). Median hospital length of stay was 6 days (mean 10.8 days).

**Conclusions:**

RSV infection is associated with the need for extended hospital stay, ICU care and mortality in adults of all ages with chronic underlying conditions. Presenting signs and symptoms are nonspecific, co-infections occur, and patients often receive antibiotics and anti-influenza therapy. There is need for ongoing research and development of RSV prophylaxis, vaccines and treatments for adults.

**Electronic supplementary material:**

The online version of this article (doi:10.1186/s12879-014-0665-2) contains supplementary material, which is available to authorized users.

## Background

RSV is the most common cause of serious lower respiratory tract infection in infants and young children, but immunity to RSV is incomplete and infections recur throughout life [1]-[10]. In adults, RSV was long thought to cause primarily mild upper respiratory tract infections. Estimates of adult hospitalization, morbidity and mortality attributable to RSV have varied, likely due to a lack of routine testing for respiratory viruses in adult acute respiratory illness and the poor sensitivity of many test methods when performed [11]. Over the last several decades, however, studies have shown that RSV causes more severe infection in the elderly, and in adults with immune compromising and chronic cardiopulmonary conditions [3],[4],[9],[12]-[19]. There now is growing evidence that rates of health care utilization, hospitalization, morbidity and mortality among adults with RSV infection may be similar to those observed with influenza infection [9],[20]-[25].

More recent studies also suggest that RSV causes greater morbidity in younger and healthier adult populations than previously appreciated [4],[26]-[28]. A prospective study of RSV infections in previously healthy working adults aged 18–60 found that 26% had LRTI symptoms and 38% required time away from work during their illness [4]. In studies of previously healthy military recruits, 11-14% with respiratory symptoms were found to have RSV and most were unwell enough to require time away from duty [26],[27]. Previously healthy adults have also been shown to have altered airway reactivity for weeks following RSV infection [29].

In order to better understand the burden of adult disease due to RSV, we undertook a retrospective study of hospitalization due to RSV infection in adults in four acute care hospitals in Toronto, Canada, during a winter season in which routine testing for RSV was added to all specimens submitted for respiratory virus testing.

## Methods

### Study location and design

A retrospective case series was conducted at four tertiary care adult teaching hospitals in the University of Toronto system: the three acute care hospitals of the University Health Network: Toronto General Hospital, Toronto Western Hospital, and the Princess Margaret Cancer Centre; and the Mount Sinai Hospital. Together these hospitals have 1208 beds for adults, providing 412,000 adult patient care days annually.

### Virus identification

A single laboratory provides microbiology service for these hospitals. During the 2012–2013 winter season, qualitative real-time reverse transcriptase polymerase chain reaction (RT-PCR) testing for influenza and RSV was performed using the Simplexa Flu A/B & RSV assay (Focus Diagnostics, Cypress, CA). Specimens from ICU patients, and all bronchoscopy specimens were also tested for respiratory viruses using the Seeplex RV15 ACE detection kit (RV15; Seegene, South Korea) panel at the Ontario Public Health Laboratory. At Mount Sinai Hospital, all patients admitted between October 15, 2012 and March 31, 2013 with either a diagnosis of a respiratory infection or symptoms including fever and cough had nasopharyngeal (NP) swabs submitted for RSV and influenza testing; at the remaining three hospitals, testing for the presence of respiratory viruses was at the discretion of attending physicians. All other laboratory testing was at the discretion of attending physicians.

### Study population

The microbiology laboratory database was used to identify all patients ≥ 18 years of age admitted to any of the four hospitals who had a specimen in which RSV was identified between September 2012 and June 2013.

Cases were excluded if RSV infection was judged to be incidental to hospital admission: that is, if patients were admitted either with a non-respiratory, non-cardiovascular diagnosis that was unrelated to RSV infection, or if their respiratory symptoms were not acute and were adequately explained by another diagnosis. Patients were also excluded if they had possible or definite healthcare associated disease: that is, if they had a negative specimen within three days of admission followed by a later positive specimen, or if they developed symptoms which led to NP swab testing more than 7 days after admission.

### Data collection

Electronic and paper medical charts were reviewed and information was collected for each patient on baseline characteristics, comorbid illness, medications (including steroids, immunosuppressive medications, and recent chemotherapy), presenting symptoms and signs, treatments, and outcomes including complications, respiratory failure requiring mechanical ventilation, need for ICU, hospital length of stay and mortality.

### Statistical analysis

All data entry and analysis was performed in SAS, version 9.3 for PC. Data were entered in duplicate and cleaned prior to analysis. Comparison of proportions was performed using chi-square and Fisher’s exact tests, as appropriate, and comparisons of continuous variables performed using Wilcoxon rank sum tests. P-values of ≤0.05 were considered significant.

The University Health Network and Mount Sinai Hospital research ethics boards granted approval of this study.

## Results

### Population

From September 2012 to June 2013, ninety-five hospitalized patients with a positive test for RSV were identified. Six patients met criteria for hospital-acquired infection (positive specimens obtained a median of 20.5 days, range 12–23 days after admission) and three patients presented with RSV infections unrelated to the reason for hospitalization (1 each admitted for reactivation of tuberculosis, pulmonary embolism, and lung cancer with hemoptysis).

**Figure 1:**
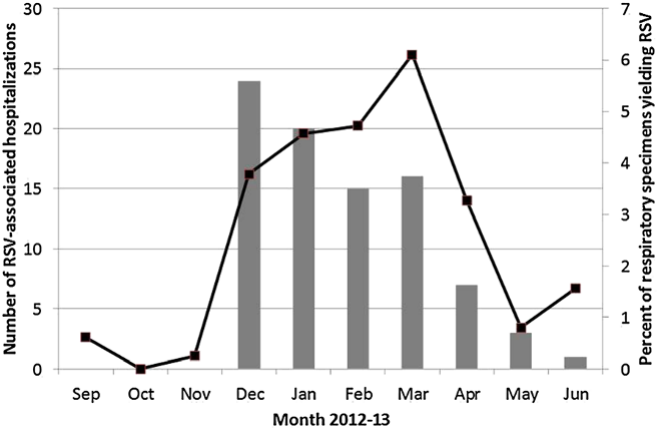
**Timing of admission for RSV patients hospitalized with acute respiratory illness (N = 86), 2012–2013 winter season.** Solid grey bars show the number of RSV-associated hospitalizations; black line shows percentage of submitted respiratory specimens positive for RSV.

Thus, eighty-six adult patients were identified with RSV infection requiring hospitalization and are included in the analysis. Hospital admission associated with RSV infection was most commonly seen from December to April, during which time >3% of all specimens submitted for testing were positive for RSV (Figure 1).

Baseline characteristics of included patients are detailed in Table 1. Their median age was 74 years (range 19–102) and 56% were female. Eighty-three (97%) had chronic underlying medical conditions, of whom 27 (31%) were immunosuppressed (15% due to disease, 59% due to therapy, and 26% due to both disease and therapy). Immunosuppressed patients tended to be younger (median age 62 years v. 81 for immunocompetent patients), although the prevalence of immunosuppression among those younger than age 65 (8/29 or 28%) was similar to that in older adults 65 (19/57 or 33%).

**Table 1:** **Baseline characteristics, signs and symptoms** ^**1**^ **of 86 patients hospitalized with RSV infection, 2012/13 winter season**

Baseline characteristic	Number (%)
Age, median (range)	74 years (range 19–102)
Female gender	48 (56%)
Institutionally-acquired^2^	14 (16%)
Underlying illness (any)	83 (97%)
Lung disease	31 (36%)
Cardiac disease	45 (52%)
Immunosuppression^3^	27 (31%)
Diabetes mellitus	27 (31%)
Known smoker	10 (12%)
Presenting symptoms/signs	
Cough	73 (85%)
Dyspnea	68 (79%)
Sputum production	54 (63%)
Weakness	43 (50%)
Fever (T > 38.0°C) in emergency department	41 (48%)
Wheezing	33 (38%)
Lethargy/malaise	27 (31%)
Runny nose or nasal congestion	19 (22%)

^1^Symptoms present in > 20% of patients are shown in table; less common symptoms included: sore throat in 16 (19%), altered LOC 16 (19%), chest pain 15 (17%), nausea 15 (17%), vomiting 14 (16%), loss of appetite 14 (16%), diarrhea 11 (13%), dizziness 11 (13%). Abdominal pain, headache, arthalgias and myalgias were reported in < 10% of patients.

^2^Institutions included nursing homes (8), retirement homes (3), rehabilitation or convalescent care facilities (2), and mental health hospitals (1).

^3^Immunosuppressed patients included those with hematologic malignancy, solid tumor on chemotherapy at time of RSV diagnosis, organ transplant, vasculitis, lupus, inflammatory bowel disease, or pulmonary fibrosis on immunosuppressive medications at time of RSV diagnosis.

### Signs and symptoms

The median duration from symptom onset to admission was 4 days (range 0–30, interquartile range 2–6.75 days). The most common presenting signs and symptoms were cough, shortness of breath, sputum production, weakness, fever, and wheezing (Table 1). The most common reasons for presentation to hospital recorded in triage or ED consultation notes were shortness of breath in 55 (64%) patients and cough in 38 (44%) patients. The cough was described as productive in 20 (23%) patients.

### Outcomes

Lower respiratory tract complications were diagnosed in 45 (52%) patients (Table 2), with 5 (6%) diagnosed with an acute exacerbation of asthma, 11 (13%) with an acute exacerbation of COPD, and 34 (40%) with pneumonia. Twenty-six (30%) of patients had radiographic abnormalities consistent with pneumonia. There was variability in radiographic appearance of pneumonia including unifocal infiltrates in 18 (21%) patients, multifocal infiltrates in 8 (9%) patients, and lobar consolidation in 11 (13%) patients. Twenty-two cardiovascular complications were diagnosed in 19 (22%) patients, including CHF exacerbation in 12 (14%), new arrhythmia in 7 (8%), stroke in 2 (2%), and myocardial infarction in 1 (1%).

**Table 2:** **Complications and outcomes of 86 patients hospitalized with RSV infection, 2012/13 winter season**

Complication/outcome	Number (%)
Lower respiratory tract complications^1^	45 (52%)
Cardiovascular complications^2^	19 (22%)
Pneumonia^3^	34 (40%)
Confirmed radiologically	26 (30%)
Unifocal infiltrate	18 (21%)
Multifocal infiltrates	8 (9%)
Lobar consolidation	11 (13%)
Co-pathogen identified^4^	11 (13%)
Viral^5^	2 (2%)
Bacterial^6^	9 (11%)
Need for intensive care	13 (15%)
Need for invasive mechanical ventilation	8 (9%)
In hospital mortality	5 (6%)
Median time to death (range)	6 days (2–52 days)
Median hospital length of stay (range)	6 days (1–140 days)

^1^Lower respiratory tract complications included exacerbation of COPD (n = 11) or asthma (n = 5), or pneumonia (n = 34).

^2^Cardiovascular complications included new arrhythmia (n = 7), CHF exacerbation (N = 12), myocardial infarction (n = 1), and stroke (n = 2).

^3^Pneumonia as diagnosed by treating physicians.

^4^Co-pathogen identified in nasopharyngeal swab, sputum or bronchoscopy specimen culture within 5 days of admission. No patients had concomitant bacteremia.

^5^Viral co-pathogens included influenza A (n = 1), and both influenza B and CMV in another patient.

^6^Bacterial co-pathogens included *Haemophilus influenzae (n =* 3), methicillin-sensitive *Staphylococcus aureus* (n = 1), *Streptococcus pyogenes* (n = 1), *Streptococcus pneumoniae* (n = 2), methicillin-resistant *Staphylococcus aureus* (n = 1), and *Pseudomonas aeruginosa* (n = 1).

No patients had concomitant bacteremia. Eleven (13%) patients had potential bacterial (n = 9; 11%) or viral (n = 2; 2%) co-pathogens identified from NP swab, sputum or bronchoscopy specimen cultures (Table 2). Bacterial co-pathogens were identified in 5/34 (15%) patients with and 4/52 (8%) of patients without clinician- diagnosed pneumonia. Antibiotics were prescribed to 78% of patients and anti-influenza medications were prescribed to 36%. Antibiotic prescription was significantly more common in those with a diagnosis of pneumonia (34/34 (100%) v. 33/52 (64%), p = <0.01), and somewhat more likely in patients for whom a potential co-pathogen was identified (11/11 (100%) v. 56/75 (75%), p = 0.06). Immunosuppressed patients were more likely to be prescribed antibiotic therapy (26/27 (96%) v. 41/59 (70%), p = 0.01). Ribavirin was prescribed for eight of 27 (30%) immunosuppressed patients, and no immunocompetent patients.

ICU care was required in 15% of patients and 9% required invasive mechanical ventilation. In hospital mortality was 6% (5/86), and the causes of death were documented as respiratory failure secondary to RSV infection (n = 1), aspiration pneumonia (n = 1), cardiac arrest due to vertebrobasilar stroke and aspiration pneumonia (n = 1), and multiorgan failure (n = 2). Time from admission to death in these 5 patients was 3, 6, 7, 12 and 53 days. Mean and median hospital LOS was 10.8 (SD 16.7) and 6 days (range 1–140).

There was no association found between advanced age, underlying cardiac or lung disease, or immunosuppression with the need for ICU care or development of pneumonia. Need for ICU care was associated with greater mortality, with deaths in 3/13 (23%) of patients requiring ICU care compared to 2/73 (3%) not requiring ICU (p = 0.01). Need for invasive MV was also associated with mortality, with deaths in 3/8 (40%) of patients requiring invasive MV compared to 2/78 (3%) of other patients (p = 0.01). None of advanced age, underlying cardiac or lung disease, immunosuppression, pneumonia, or identification of a bacterial co-pathogen were associated with mortality.

## Discussion

This study contributes to the growing body of evidence documenting that RSV is a serious pathogen in adults. In our cohort, adult hospitalization associated with RSV infection was not uncommon, and it was associated with a substantial rate of complications and mortality. Although the majority of adults were older, more than one-third were less than 65 years of age.

To complement the findings of this study, we conducted a literature search using MEDLINE (1946- December 2013), EMBASE (1947- December 2013), and PubMed (through December 31, 2013) databases. The references for each publication were reviewed to search for additional studies. After excluding studies with nosocomial cases, those focused specifically on ICU patients, or those that did not specifically describe RSV infections, we identified six previous cohort studies of RSV hospitalization in adults (Table 3) [9],[20],[21],[25],[28],[30]. One of these cohorts had data in more than one publication [9],[31].

**Table 3:** **Published studies of adult patients hospitalized with acute respiratory symptoms and RSV infection**

Reference	Years of study	Type of study	Geographic location	Population	Means of diagnosis
Lee [25]	2009-2011	Retrospective cohort	Hong Kong, China	607 adults (≥ 18 years) with acute respiratory infection	Immunofluorescence assay, nasopharyngeal aspirates
Widmer [21]	2006-2009	Prospective cohort	Tennessee, USA	31 adults ≥ 50 years with respiratory symptoms or non-localizing fever	RT-PCR, frozen nasal and throat swabs
Falsey [9]	1999-2003	Prospective cohort	New York, USA	132 adults ≥ 65 years with underlying cardiopulmonary disease and acute respiratory symptoms	Culture and RT-PCR of nasopharyngeal specimens; and acute and convalescent serology
Dowell [28]	1990-1992	Prospective cohort	Ohio, USA	47 community dwelling adults (≥ 18 years) with pneumonia	Acute and convalescent serology
Falsey [20]	1989-1992	Prospective cohort	New York, USA	145 community dwelling adults ≥ 65 years with acute cardiopulmonary conditions or influenza-like illness	Antigen detection, culture on nasopharyngeal specimens; acute and convalescent serology
Vikerfors [30]	1971-1980	Retrospective cohort	Orebro, Sweden	57 adults (> 16 years) with pneumonia	Immunofluorescence assay on nasopharyngeal secretions; acute and convalescent serology

*Abbreviations*: RT-PCR, reverse transcriptase PCR; USA, United States of America.

Despite substantial differences in time, geography, and means of diagnosis of RSV infections, the combined results provide a consistent and cohesive view of the characteristics of these patients and their disease (Tables 3 and 4). The majority of patients in these studies were older, and both underlying pulmonary and cardiac disease were common. There were however, differences in the distribution of underlying conditions. For instance, the proportion of immunosuppressed patients was noticeably greater in our study and in that of Widmer et al. than that of Lee et al [21],[25]. This difference may be due to differences in patient populations cared for in study hospitals, definitions of immunosuppression, or criteria for RSV testing. In our study subjects were considered immunosuppressed if they had a hematologic malignancy, solid tumor on chemotherapy, chronic renal failure, liver cirrhosis, or if they were on immunosuppressive therapy (see Table 1). Lee et al. do not describe the conditions considered immunocompromising, and Widmer et al. appear to have included all malignancies, as well as splenectomy and HIV/AIDS [21],[25].

**Table 4:** **Characteristics and outcomes of adult patients hospitalized with acute respiratory symptoms and diagnosed with RSV as reported in published studies**

Study	This study (all adults)	Lee 2013 (all adults)	Widmer, 2012 (50+ yrs)	Falsey, 2005 (65+ yrs)	Dowell, 1996 (all adults)	Falsey, 1995 (65+ yrs)	Vikerfors, 1987 (all adults)
Age	Median 74 yrs IQR 62-85	Median 80 yrs IQR 68-86	Median 68 yrs IQR 56-78	Mean 76 yrs SD 13	Mean 61 yrs range 21-89	Mean 80 yrs SD 8	Median 75 yrs
Underlying illness							
Any	97%	87%	-	-	-	-	56%
Lung disease	36%	36%	68%	54%	>65%*	43%	14%
Cardiac disease	52%	-	48%	58%	>49%*	63%	-
Immunosuppressed	31%	14%	61%		-	-	-
Complications							
LRT complication	52%	72%	-	-	-	63%‡	-
Pneumonia†	30%	42%	-	31%	40%	48%	-
Cardiovascular	22%	14%	-	13% (CHF)	-	20% (CHF)‡	-
Co-pathogen identified	13%	13%	0	15% sputum	14%	17% sputum	25%
				3% blood		4% blood	
Hospital course							
Required ICU	15%	-	10%	15%	21%	18%	-
Required MV	9%	2%	3%	13%	7%	10%	-
Length of stay	Median 6d	Median 7d	Median 3d	Mean 14d	Mean 9d	Mean 16d	-
Case fatality rate	6% (in hospital)	9% (30d)	7% (in hospital)	8% (not specified)	-	10% (in hospital)	0

*65% of patients had COPD, 44% had asthma; 49% had coronary artery disease; 37% congestive heart failure.

†Confirmed by CXR, or pulmonary infiltrates present on CXR.

‡Derived from principal discharge diagnoses.

*Abbreviations*: LRT = lower respiratory tract, ICU = intensive care unit, MV = mechanical ventilation.

Adult patients with RSV infection often experience symptoms of nasal congestion, productive cough, and low-grade fever that may or may not be followed within a few days by dyspnea and wheezing. Rates of runny nose or nasal congestion (22% versus mean of 58%) and wheezing (38% versus mean of 64%) were lower in our study compared to other studies [4],[9],[20],[21],[25],[28]. This may be a limitation of our retrospective study design as treating clinicians may have failed to document these symptoms. Rates of other symptoms were similar to those observed in previous studies in which they could not facilitate differentiation from other respiratory illnesses.

Adult patients with RSV infection often present with an exacerbation of underlying cardiac or pulmonary disease, such as asthma, COPD, or CHF [32]-[34]. In our study, the admitting diagnosis for 23 (27%) patients was recorded as an exacerbation of underlying lung disease or cardiac disease.

Lower respiratory tract complications including pneumonia were common in our study and in previous studies. Radiographic appearance of pneumonia associated with RSV infection has been noted to be variable, ranging from the absence of detected abnormality to small and unilateral pulmonary infiltrates, symmetrical or asymmetrical bilateral pulmonary infiltrates, or lobar consolidation [7],[20],[31]. This variability was also seen in our study.

In our cohort potential co-pathogens were identified in 13% of cases, similar to the rate observed in other published studies [9],[25],[28]. Vikerfors et al. observed much higher rates of co-pathogens, but their positive cultures for co-pathogens included results from nasopharyngeal samples rather than sputum or blood cultures as collected in other studies [20],[25],[30],[32]. Widmer et al. report no co-infections in their cohort, although it is unclear whether pathogens present in non-sterile site specimens would have met criteria for co-infections in their patients [21].

Requirement for invasive mechanical ventilation, ICU care, mortality rates, and hospital lengths of stay were substantial and consistent across studies [9],[20],[21],[25],[28],[30]. Vikerfors et al. observed a lower case fatality rate, which may be a result of the lower prevalence of underlying illness in their cohort [30].

Antibiotics were commonly prescribed in our cohort, as was the case in two other large studies of adult patients hospitalized with RSV [20],[25]. While co-infections have been noted to occur, these numbers highlight the challenges of optimal use of antibiotics when viral respiratory infections have been diagnosed, but bacterial pathogens not identified. Due to difficulties with diagnosis of co-existent bacterial infections, the most effective way to reduce this overuse may be through prevention of RSV infection via enhanced infection control measures, development of effective prophylaxis or, as noted in a recent commentary, vaccination [35].

Advantages of our study are that we have included adults of all ages, and that testing was performed by RT-PCR. Previous testing for RSV has been done using antigen immunoassays or culture, which lack sensitivity, or using acute and convalescent serology, which cannot identify fatal cases, and may also lack sensitivity in immunocompromised patients or the frail elderly [11],[36]-[38]. Most previously published studies using RT-PCR have also included only older adults [9],[21],[25]. Our study demonstrates that a substantial fraction of adult patients with RSV infection are not elderly; however, virtually all did have chronic underlying illnesses.

This study has several limitations. It took place in only one geographic location during only one season, and may therefore not be generalizable to other seasons or locations. Testing of patients was not systematic in all hospitals, and we may have failed to identify some infected patients. Our use of RT-PCR for diagnosis may have resulted in some false positive tests, although the Simplexa Flu A/B & RSV assay (Focus Diagnostics, Cypress, CA) used for diagnosis has good performance characteristics and high specificity [39],[40]. Finally, RSV infection may have been incidental and the requirement for hospital admission due to other causes; this may be a particular issue in immunocompromised patients, who may shed RSV for prolonged periods of time [18].

## Conclusion

In conclusion, RSV infection in hospitalized adults is associated with substantial morbidity and the need for extended hospital stay, ICU care and mortality in adults of all ages with chronic underlying conditions. The data produced from this study are useful in supporting existing data from other areas, and reinforce that RSV infection in hospitalized adults is a global issue. There is need for ongoing research and development of effective RSV prophylaxis, vaccines and treatments.

## Authors’ original submitted files for images

Below are the links to the authors’ original submitted files for images. Authors’ original file for figure 1
