# Supplementary figures and images for: Respiratory syncytial virus infection-associated hospitalization in adults: a retrospective cohort study
Source: BMC Infect Dis. 2014 Dec 13;14:665. doi: 10.1186/s12879-014-0665-2 (PMC4269936; doi:10.1186/s12879-014-0665-2)

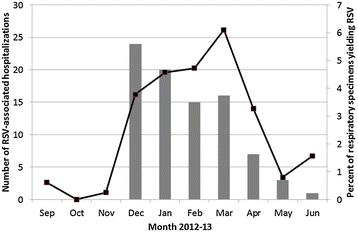

Supplement: Supplementary file 1 — Authors’ original file for figure 1 [file 12879_2014_665_MOESM1_ESM.gif]
